# Functional Alterations Involved in Increased Bleeding in Hereditary Hemorrhagic Telangiectasia Mouse Models

**DOI:** 10.3389/fmed.2022.871903

**Published:** 2022-05-19

**Authors:** Cristina Egido-Turrión, Elisa Rossi, Claudia Ollauri-Ibáñez, María L. Pérez-García, María A. Sevilla, José María Bastida, José Ramón González-Porras, Alicia Rodríguez-Barbero, Carmelo Bernabeu, José M. Lopez-Novoa, Miguel Pericacho

**Affiliations:** ^1^Department of Physiology and Pharmacology, Universidad de Salamanca, Salamanca, Spain; ^2^Institute for Biomedical Research of Salamanca (IBSAL), Salamanca, Spain; ^3^Université de Paris, Innovative Therapies in Haemostasis, INSERM, Paris, France; ^4^Department of Internal Medicine, Complejo Asistencial Universitario de Salamanca (CAUSA)-SACYL, Salamanca, Spain; ^5^Department of Hematology, Complejo Asistencial Universitario de Salamanca (CAUSA)-SACYL, Salamanca, Spain; ^6^Centro de Investigaciones Biológicas Margarita Salas, Consejo Superior de Investigaciones Científicas (CSIC), Madrid, Spain

**Keywords:** bleeding, hemostasia, Hereditary Hemorrhagic Telangiectasia (HHT), ALK1 (ACVRL1), endoglin (CD105)

## Abstract

Hereditary Hemorrhagic Telangiectasia (HHT) is an autosomal-dominant genetic disorder involving defects in two predominant genes known as endoglin (*ENG*; HHT-1) and activin receptor-like kinase 1 (*ACVRL1/ALK1*; HHT-2). It is characterized by mucocutaneous telangiectases that, due to their fragility, frequently break causing recurrent epistaxis and gastrointestinal bleeding. Because of the severity of hemorrhages, the study of the hemostasis involved in these vascular ruptures is critical to find therapies for this disease. Our results demonstrate that HHT patients with high bleeding, as determined by a high Epistaxis Severity Score (ESS), do not have prolonged clotting times or alterations in clotting factors. Considering that coagulation is only one of the processes involved in hemostasis, the main objective of this study was to investigate the overall mechanisms of hemostasis in HHT-1 (*Eng*^+/−^) and HHT-2 (*Alk1*^+/−^) mouse models, which do not show HHT vascular phenotypes in the meaning of spontaneous bleeding. In *Eng*^+/−^ mice, the results of *in vivo* and *in vitro* assays suggest deficient platelet-endothelium interactions that impair a robust and stable thrombus formation. Consequently, the thrombus could be torn off and dragged by the mechanical force exerted by the bloodstream, leading to the reappearance of hemorrhages. In *Alk1*^+/−^ mice, an overactivation of the fibrinolysis system was observed. These results support the idea that endoglin and Alk1 haploinsufficiency leads to a common phenotype of impaired hemostasis, but through different mechanisms. This contribution opens new therapeutic approaches to HHT patients' epistaxis.

## Introduction

Hereditary Hemorrhagic Telangiectasia (HHT), also known as Osler-Weber-Rendu disease, is an autosomal-dominant genetic disorder involving genetic defects in two predominant genes: *ENG*, codifying for endoglin (HHT-1 type) and *ACVRL1/ALK1*, codifying for activin receptor-like kinase 1 (HHT-2 type); and, much less frequently, in two other genes, such as *MADH4/SMAD4* (JP-HHT) or *BMP9/GDF2* (HHT-5) (1). HHT has an estimated prevalence of at least 1/5,000 and is characterized by the presence of multiple dermal, mucous and visceral arteriovenous malformations (AVMs) in lungs, liver, gastrointestinal tract and nervous system (2–4), as well as recurrent and severe hemorrhages (5, 6).

Generally, nosebleeds, also known as epistaxis, are not only the first symptom to manifest the pathology, but also the most frequent, since patients usually bleed more than once a week. This frequency increases with age and 12% of diagnosed cases have more than one bleeding per day (7). The intensity can vary from a few drops to events that last up to 10 min, and although nasal packing is often used to control acute bleeding, even though it may cause re-bleeding, there are no well-designed studies on the first-line treatment of acute epistaxis (8, 9). The combination of the high frequency linked to the severity of the bleeding leads 50% of patients to develop iron deficiency anemia (10). Usually, bleeds are so uncontrolled that administration of iron supplement is not enough to reverse anemia, so it is not uncommon to treat patients with repeated blood transfusions (11, 12). Both bevacizumab, an antiangiogenic monoclonal antibody that selectively targets Vascular Endothelial Growth Factor (VEGF) (13), and thalidomide, a potent immunosuppressive and antiangiogenic agent (14) could be used to treat HHT patient hemorrhages but with long-term side effects (15). For these reasons the study of hemostasis in HHT mouse models could contribute to better understand the pathology and improve treatments.

It is widely recognized that the origin of HHT bleeding is the fragility of the mucocutaneous telangiectases which tend to break, causing recurrent epistaxis and gastrointestinal bleeding. However, it is unclear why the hemostatic system does not properly control these bleeds. Intriguingly, despite the severity of the bleeds, HHT has not been described as a disease that presents deficiencies in any of the clotting factors (16) and no systematic studies on the rest of the processes related to the control of bleeding have been performed. A previous pioneer study demonstrated that endoglin (Eng), a protein expressed on endothelial cells and in heterozygous condition in HHT-1 patients, is able to bind the integrin αIIbβ3 present on platelets via its RGD motif (17). This binding contributes to platelet interaction with activated endothelial cells and increases platelet resistance to shear, whereas loss of Eng seems to interfere with these platelets-endothelium interactions (17), confirming that Eng can display a role as an adhesion molecule in addition to its well-known function as TGFβ co-receptor (18).

In this study, after confirming with HHT-1 and HHT-2 patients that increased bleeding is not associated with alterations in prothrombin time (PT), activated partial thromboplastin time (aPTT) or coagulation factors, we used the most common genetic models of HHT, namely *Eng*^+/−^ for HHT-1 and *Alk1*^+/−^ for HHT-2 (19), as well as their *Wildtype* (*WT*) littermates (*Eng*^+/+^ and *Alk1*^+/+^, respectively) as controls to analyze the overall process of hemostasis. Our hypothesis is that, while the high frequency of bleeds seems clearly due to rupture of fragile vessels resulting from impaired angiogenesis, the severity of bleeds may be due to alterations in the hemostatic process. Taking into account that Eng and ALK1 are mostly expressed in the endothelium, we have focused on the processes where the endothelium is most relevant, especially in thrombus stabilization, where endoglin may play an important role.

## Materials and Methods

### Materials

If not specifically mentioned, all the reagents and vasoactive substances were from Sigma-Aldrich (Merck Life Science S.L.U, Darmstadt, Germany).

### Patients and Epistaxis Severity Score

In total, 36 HHT patients, 5 with HHT-1 and 31 with HHT-2, participated in the study. Relatives not affected by the disease were recruited as controls for the study. All of them signed an informed consent form. The study was approved by the Local Ethical Committee. Epistaxis Severity Score were calculated for all participants in the study as previously described (20).

### Coagulation Studies

Venous blood samples were drawn into commercial buffered 3.2% sodium citrate for coagulation studies. Coagulation basic studies consisted of prothrombin time (PT) and activated partial thromboplastin time (APTT) and were measured according to international recommendations (21). Factor coagulant activity (Factor:C) was measured on a BCS XP System (Siemens, Healthcare Diagnostics, Erlangen, Germany) by modification of the one-stage clotting method with the use of factor deficient or depleted plasmas. Coagulation time by BCS XP was calibrated with standard human plasma. The normal ranges were considered to be 60–120%. Von Willebrand (vW) Factor activity was analyzed using latex particles surface-coated with recombinant GPIb (Siemens, Healthcare Diagnostics, Erlangen, Germany) (22, 23).

### Animal Models: *Eng^+/−^* (HHT-1), *Alk1^+/−^* (HHT-2), and *ENG^+^*

All animal protocols were performed with approval of the Committee for the Care and Use of Animals of the University of Salamanca (Spain), project #305 and complied with the current guides of the European Union and the US Department of Health and Human Services for the Care and Use of Laboratory Animals. Generation of *Eng*^+/−^ and *Alk1*^+/−^ mice has been described previously (24, 25). The animals were a generous gift from Michelle Letarte (Hospital for Sick Children, Toronto, Canada) and Peter ten Dijke (Leiden University, Netherlands), respectively. Both transgenic lines were backcrossed with C57BL/6J mice for more than ten generations. Of note, the genetic background of these HHT animal models do not show the characteristic bleeding phenotype due to fragile telangiectases as in other strains (26). Routine genotyping of DNA isolated from mouse tail biopsies was performed by PCR using the primers previously reported (25, 27). The generation and characteristics of the transgenic mice ubiquitously overexpressing human endoglin (*ENG*^+^) was performed by microinjection of a pCAGGS vector containing the complementary DNA sequence (cDNA) of endoglin in the fertilized egg of CBAxC57BL/6J mice, as previously described (28). *WT* littermates for each model were used as controls.

For animal anesthesia, 2% isoflurane in oxygen was used. During recovery from the anesthesia, heat was provided and, when necessary, a dose of buprenorphine (0.05 mg/kg) was subcutaneously administered. Animals were sacrificed by CO_2_ inhalation or cervical dislocation, depending on the experiment requirements.

### Tail-Bleeding Assay

Isoflurane-anesthetized mice were placed in prone position. Their tails were transected at ~3 mm from the tip (29, 30) (constant tail diameter 1.4 mm) and immediately immersed in PBS at 37°C. Each animal was monitored for 30 min even if bleeding ceased, in order to detect any re-bleeding. However, when mice bled continuously for more than 10 min, the experiment was interrupted so that the life of the animal was not threatened. Three parameters were analyzed: (i) first bleeding time, the period between the beginning of the hemorrhage and the first time it stopped; (ii) re-bleeding time, defined as the time of the rest of periods where the hemorrhage starts again; and (iii) total bleeding time, the sum of the time in which the bleeding is active.

### Vascular Reactivity of Thoracic Aorta Rings

To analyze the capacity for reflex vasoconstriction of the vessels of mice models, we conducted studies of vasomotor functionality in thoracic aorta arteries obtained from mice of the different genotypes, as previously described (31). Briefly, arteries were removed and placed in chilled Krebs solution (118 mM NaCl, 4.7 mM KCl, 2.5 mM CaCl_2_, 1.2 mM KH_2_PO_4_, 1.2 mM MgSO_4_, 25 mM NaHCO_3_, and 11 mM glucose). The pH of the solution after saturation with carbogen was 7.4. The arteries were carefully cleaned, cut into rings (2 mm) and aortic rings were mounted, with Krebs solution, onto a 4-channel myograph (Multiwire Myograph System, DMT, Denmark) After an equilibration period all rings were normalized and set at a resting tension of 5 mN and allowed to equilibrate for 30 min. After that, the vessels were tested for responsiveness to a hyperpotassium solution (KPSS; KCl 120 mM) and cumulative concentration–response curves to norepinephrine, serotonin and thromboxane analog U-46619 were generated in separate rings until the maximal response was consistent. Vasoconstrictor responses were expressed as a percentage of KCl-induced contractions. The cumulative concentration–response curves, were fitted to a logistic equation and analyzed with GraphPad Prism 9.0 software. We carried out statistical analysis of concentration–response curves using the extra sum-of-squares *F* test principle.

### Induction of Thrombogenic Event

Mice were anesthetized as previously described and a catheter was implanted in the jugular vein. Collagen (150 ng/g body weight; Chrono-Log, Havertown, PA, USA) and epinephrine (15 ng/g body weight) were administered intravenously and blood samples were collected from jugular vein into EDTA tubes before and after 3 min of the injection of these compounds, that lead to platelet activation, aggregation and adhesion and, consequently, a decrease in platelet count. Platelet count in these samples was performed in an automated complete blood count analyzer (AdviaTM 120 hematology; Bayer, Leverkusen, Germany). At the end of experiment, anesthetized animals were euthanized by cervical dislocation.

### Platelet Activation and Aggregation Analysis by Flow Cytometry

Murine blood samples were obtained by retroorbital puncture using citrated capillary. Whole blood samples were centrifuged at 1,300 g for 5 min and resuspended in 1 mL of Tyrode's buffer (134 mM NaCl; 20 mM HEPES; 12 mM NaHCO_3_; 5 mM glucose; 2.9 mM KCl; 1 mM MgCl_2_; 0.34 mM Na_2_HPO_4_; pH 7.4). Due to the limited volume of blood samples obtained, flow cytometry was used to test platelets activation and aggregation as previously reported (32). Aliquots of diluted blood (30 μL) were incubated for 15 min at room temperature with the appropriate anti-mouse monoclonal antibody: APC-conjugated rat anti-integrin alpha IIb (GPIIb, CD41) (6.7 μg/mL; clone MWReg30; eBioscience, Thermo Fisher Scientific, Waltham, MA, USA) and PE-labeled rat anti-mouse integrin αIIbβ3 (GPIIbIIIa, CD41/61) (25 μg/mL; clone JON/A; Emfret Analytics, Würzburg, Germany) were used to detect platelets activation. PE-labeled CD9 and FITC-labeled CD9 (5 μg/mL; clone MZ3; BioLegend, San Diego, CA, USA) were used to visualize aggregation and the double positive population was considered the aggregated platelets. Thrombin (1 U/mL) was added when necessary. To remove excess antibody, aliquots were centrifuged (2,250 g for 5 min) and resuspended in 150 μL of Tyrode's buffer. Analysis was immediately performed in a BD Accury™ C6 flow cytometer (BD Biosciences, Franklin Lakes, NJ, USA).

### Primary Culture of Mouse Lung Endothelial Cells (MLEC)

Mouse Lung Endothelial Cells (MLEC) were isolated from the different mice described above (33). MLECs were grown in Advanced-DMEM F-12 medium (Thermo Fisher Scientific, Waltham, MA, USA) supplemented with 20% FBS, 50 U/mL penicillin–streptomycin, 2 mM glutamine, 30 μg/mL endothelial cell growth supplement (ECGS) (Generon, Slough, UK) and 100 μg/mL bovine heparin in Petri dishes coated with 0.1% gelatin and supplemented with 0.01% collagen (Corning, Corning, NY, USA) and 0.01% fibronectin. Cells were maintained in 90% RH, 5% CO_2_ atmosphere at 37°C. MLECs between passages 3 and 6 were used for the experiments.

### Obtention of Platelets From Human Blood Samples

Citrated blood samples were obtained from healthy donors, who had not taken any medication for at least 10 days. Whole blood was centrifuged at 100 g for 20 min to obtain platelet-rich plasma (PRP). PRP was washed twice by diluting with washing buffer (103 mM NaCl, 5 mM KCl, 2 mM CaCl_2_, 1 mM MgCl_2_, 5 mM glucose and 36 mM citric acid; pH 6.5) supplemented with platelet activation inhibitor PGE1 (2 × 10^−7^ M) and the ADP scavenger apyrase (1 U/mL), followed by centrifugation for 12 min at 1,240 g. Platelets were finally resuspended at 2.5 × 10^8^ platelets/mL in Walsh's buffer (137 mM NaCl, 20 mM PIPES, 5.6 mM dextrose, 1 g/L BSA, 1 mM MgCl_2_, 2.7 mM KCl, 3.3 mM NaH_2_PO_4_; pH 7.4). Finally, washed platelets were stained with 1 μM calcein (Thermo Fisher Scientific, Waltham, MA, USA). Prior to any experimental procedure, platelets were typically left at room temperature for 30 min. Under resting conditions, platelets did not show activation.

### Static Platelet Adhesion Assay

MLECs were seeded in slides (Millicell EZ slide; Millipore, Burlington, MA, USA) and grown to reach a monolayer. Human-stained-platelets (10^7^ platelets/250 μL) were added on the MLECs monolayer. After an incubation for 30 min in the presence of CXCL12 (200 ng/mL; R&D Systems, Minneapolis, MN, USA), chamber wells were washed twice with PBS. Platelet adhesion was observed under a fluorescent microscope (Observer D1; Zeiss, Jena, Germany) coupled to a CCD camera (QIclick FCLR-12; Qimaging, Surrey, Canada) and quantifications were performed with ImageJ software.

### Dynamic Platelet Adhesion Assays

Microfluid devices were used to perform flow assays as previously described (34). Briefly, the channels were coated with a monolayer of MLECs. Calcein-stained platelets (2.5 × 10^8^ platelets/ml) were perfused through the channels at 2 dynes/cm^2^, allowed to adhere in the absence of flow for 10 min in presence of CXCL12. Then, the chambers were subjected to 2 dynes/cm^2^ flow for 2 min. Real-time platelet adhesion was monitored under fluorescent microscope (Observer D1; Zeiss, Jena, Germany) coupled to a CCD camera (QIclick FCLR-12; Qimaging, Surrey, Canada). Then, images were quantified by ImageJ software.

### Carotid Artery Occlusion Induced by FeCl_3_

Carotid arteries of isoflurane-anesthetized animals were exposed by a surgical opening in the ventral face of the neck. Before causing the vascular injury, a flow probe was placed around the carotid artery and carotid blood flow was recorded for 3 min using a Transonic Model TS420 flowmeter (Transonic Systems, Ithaca, NY, USA) in order to determine basal blood flow. After causing vascular damage by applying a filter paper saturated with 7.5% FeCl_3_ on top of the vessel for 1.5 min, carotid blood flow was recorded for 30 min and represented as the percentage of carotid basal blood flow. The time to carotid occlusion was determined as the time required to achieve a flow rate of <5% that remains stable for more than 5 min.

### Study of Fibrinolysis

Murine EDTA-blood samples were collected before and after 30 min, 6 h or 24 h since the intravenous administration of collagen (150 ng/g body weight; Chrono-Log, Havertown, PA, USA) and epinephrine (15 ng/g body weight) to generate a thrombogenic event and fibrinolysis system activation. Anticoagulated EDTA-blood was centrifuged and stored according to ELISA kits instructions. Plasminogen, t-PA, PAI-1 and D-Dimer levels were analyzed, respectively, by Mouse Plasminogen (PLG) ELISA kit (Cusabio, Houston, TX, USA), Mouse t-PA (Tissue-type Plasminogen Activator) ELISA kit (Elabscience, Houston, TX, USA), RayBio® Mouse PAI-1 ELISA kit (RayBiotech, Norcross, GA, USA) and Mouse D-Dimer, D2D ELISA kit (Cusabio, Houston, TX, USA) strictly following datasheet instructions. In all cases, we analyzed statistically, by two-way ANOVA, whether in each mouse model there were significant changes in the levels of the factor studied after the thrombotic event.

### Statistical Analysis

Data are expressed as the mean + standard error of the mean. All results are from at least 3 independent experiments and subjected to statistical test. Data were checked for Gaussian distribution using the D'Agostino-Pearson normality test. For normally distributed datasets, Student's *t*-test, ANOVA and two-way ANOVA was used, followed by Sidak's test for multiple comparisons. The statistical analysis of non-parametric datatests was made by the Mann-Whitney test (for two datasets) and Kruskal-Wallis test (more than two datasets). Fisher's exact test was used for the comparison of proportions when necessary. Results were considered statistical significant if *p* < 0.05 (ns: non-significant; ^*^p < 0.05; ^**^p < 0.01; ^***^p < 0.001; ^****^p < 0.0001). Graphs and statistical analysis were performed using GraphPad Prism 9 software (GraphPad software, San Diego, CA, USA).

## Results

### Increased Bleeding Is Not Associated With Alterations of Coagulation Pathways in HHT Patients

**Figure 1:**
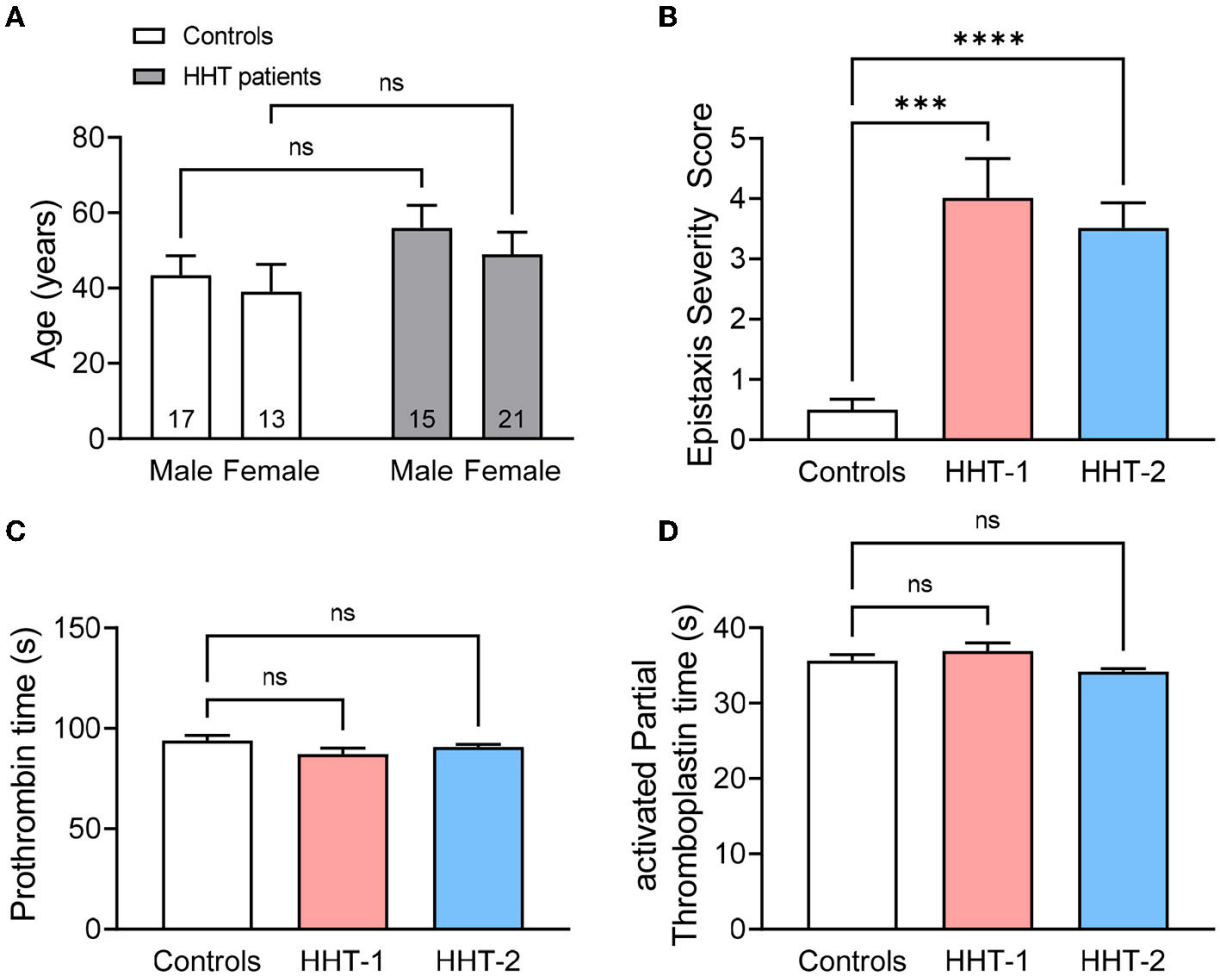
**(A)** Mean age of HHT patients (*n* = 15 males and 21 females) and its relatives unaffected by the disease that are the controls (*n* = 17 males and 13 females) of the study, disaggregated by gender. **(B)** Epistaxis Severity Score of controls (*n* = 30), HHT-1 (*n* = 5) and HHT-2 (*n* = 31) patients. **(C,D)** Prothrombin time **(C)** and activated Partial Thromboplastin time **(D)** of controls, HHT-1 and HHT-2 patients. Results were considered statistically significant if *p* < 0.05 (ns: non-significant; *: *p* < 0.05; **: *p* < 0.01; ***: *p* < 0.001; ****: *p* < 0.0001).

Epistaxis Severity Score (ESS) was used to measure the severity and frequency of epistaxis in a group of HHT-1 and HHT-2 patients, as well as in relatives not affected by the disease who are the controls of the study. The distribution of controls and patients in age and gender is shown in Figure 1A. In the patient's group there is a slightly more number of men than women and a higher mean age compared to controls, but no significant statistical differences between the two groups were found.

As expected, the ESS of HHT patients is significantly higher than that of their healthy relatives (Figure 1B). In line with previous data in the literature and Rendu's statements when first describing the disease (35), there are no differences between control subjetcs and HHT-1 or HHT-2 patients in the coagulation basic studies, neither coagulation factor activity of the intrinsic or extrinsic coagulation pathways (Figures 1C,D, Supplementary Figure S1).

### Endoglin or Alk1 Deficiency Leads to Longer Tail Bleeding Time in Mice

Having confirmed that increased bleeding in HHT patients is not due to coagulation alterations, and because dissecting other components of the hemostasis process in patients is not feasible due to ethical reasons, we decided to investigate it in murine genetic models of the disease: endoglin heterozygous mice (*Eng*^+/−^) for HHT-1 and Alk1 heterozygous mice (*Alk1*^+/−^) for HHT-2. As indicated in the methods section, the genetic background of these models do not present a bleeding phenotype due to fragile telangiectases as in other strains (26).

First, we aimed to analyze whether HHT mouse models exhibit increased bleeding when the lesion occurs in a normal vessel, rather than in a fragile vascular lesion as an HHT telangiectasia. For this purpose, we measured the tail bleeding time, which allows the analysis not only of the bleeding time of mice after wounding, but also the presence and duration of any spontaneous rebleeding.

**Figure 2:**
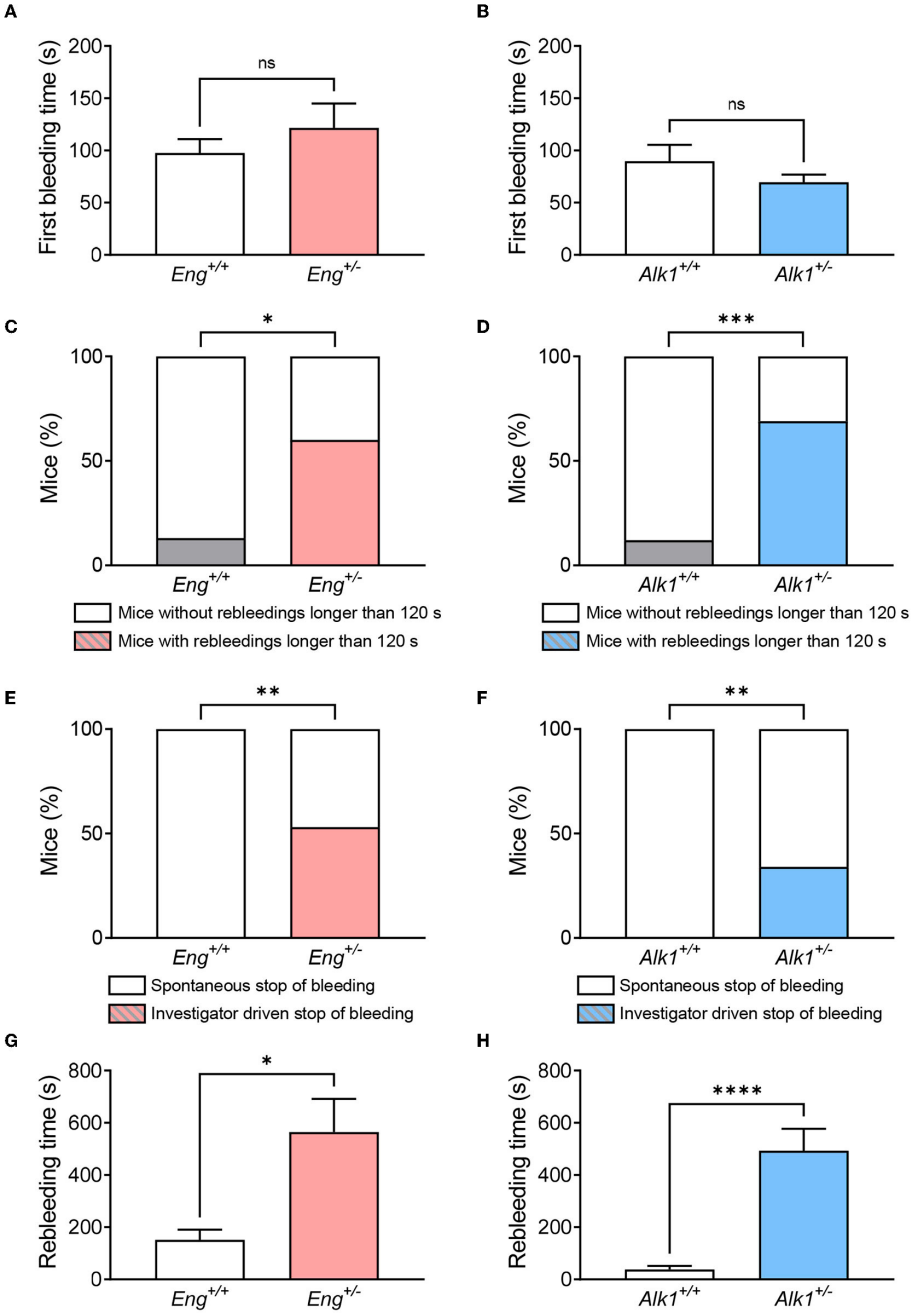
**(A,B)** Time of first bleeding after cutting the tail tip in the HHT-1 murine model (A), *Eng*^+/−^ mice (*n* = 15) and its respective controls *Eng*^+/+^ (*n* = 15); and in the HHT-2 murine model **(B)**, *Alk1*^+/−^ mice (*n* = 16) and its respective controls *Alk1*^+/+^ (*n* = 29). **(C,D)** Percentage of mice showing rebleeds longer than 2 min in the mouse model of HHT-1 **(C)** and HHT-2 **(D)**. **(E,F)** Proportion of mice that are able to stop rebleeding on their own compared to those that need investigator intervention to stop rebleeding in the animal model of HHT-1 **(E)** or HHT-2 **(F)**. **(G,H)** Rebleeding time, calculated as the sum of the time taken for the different spontaneous rebleeding times that a mouse may have during the experiment, for Eng-deficient mice and their controls **(G)**, as well as for Alk1-deficient mice and their controls **(H)**. Results were considered statistically significant if *p* < 0.05 (ns: non-significant; *: *p* < 0.05; **: *p* < 0.01; ***: *p* < 0.001; ****: *p* < 0.0001).

Although, our data show that there is no difference in the first bleeding (Figures 2A,B), when rebleeding is analyzed, the number of mice with intense rebleeds (longer than 120 s) is significantly higher in both *Eng*^+/−^ and *Alk1*^+/−^ mice with respect to their controls (Figures 2C,D). Actually, rebleeding can become so severe that in some *Eng*^+/−^ and *Alk1*^+/−^ animals the bleeding may not stop spontaneously after 30 min from injury and, thus, it must be stopped by the investigator (Figures 2E,F). For these reasons, the rebleeding time, i.e., the sum of the times of the subsequent spontaneous bleedings, is significantly longer in both *Eng*^+/−^ or *Alk1*^+/−^ mice with respect to their controls (Figures 2G,H). In conclusion, both murine models of the disease have a prolonged bleeding time suggesting an impaired hemostasis.

### Vascular Contractile Response Is Not Reduced in *Eng^+/−^* or *Alk1^+/−^* Mice

Hemostasis is firstly dependent on a vascular spasm that reduces blood flow and increments the efficacy of the following steps of hemostasis, such as platelet activation and aggregation. Thus, we analyzed in artery rings obtained from thoracic aorta of HHT-1 and HHT-2 animal models whether they presented a lower response to different vasoconstrictor agents involved in vascular spasm, such as norepinephrine, serotonin and thromboxane analog U-46619, which could explain the increased bleeding in these animals. As shown in Supplementary Figure S2, significant differences were only found with respect to the control in the response of *Eng*^+/–^ mice to serotonin (Supplementary Figure S2C). However, these mice showed an increase in the contraction response, rather than a reduction. Taken together, our results support the conclusion that the areterial contractile response is not reduced in both HHT animal models.

### Platelet Activation and Aggregation Is Not Altered in Animal Models of HHT

**Figure 3:**
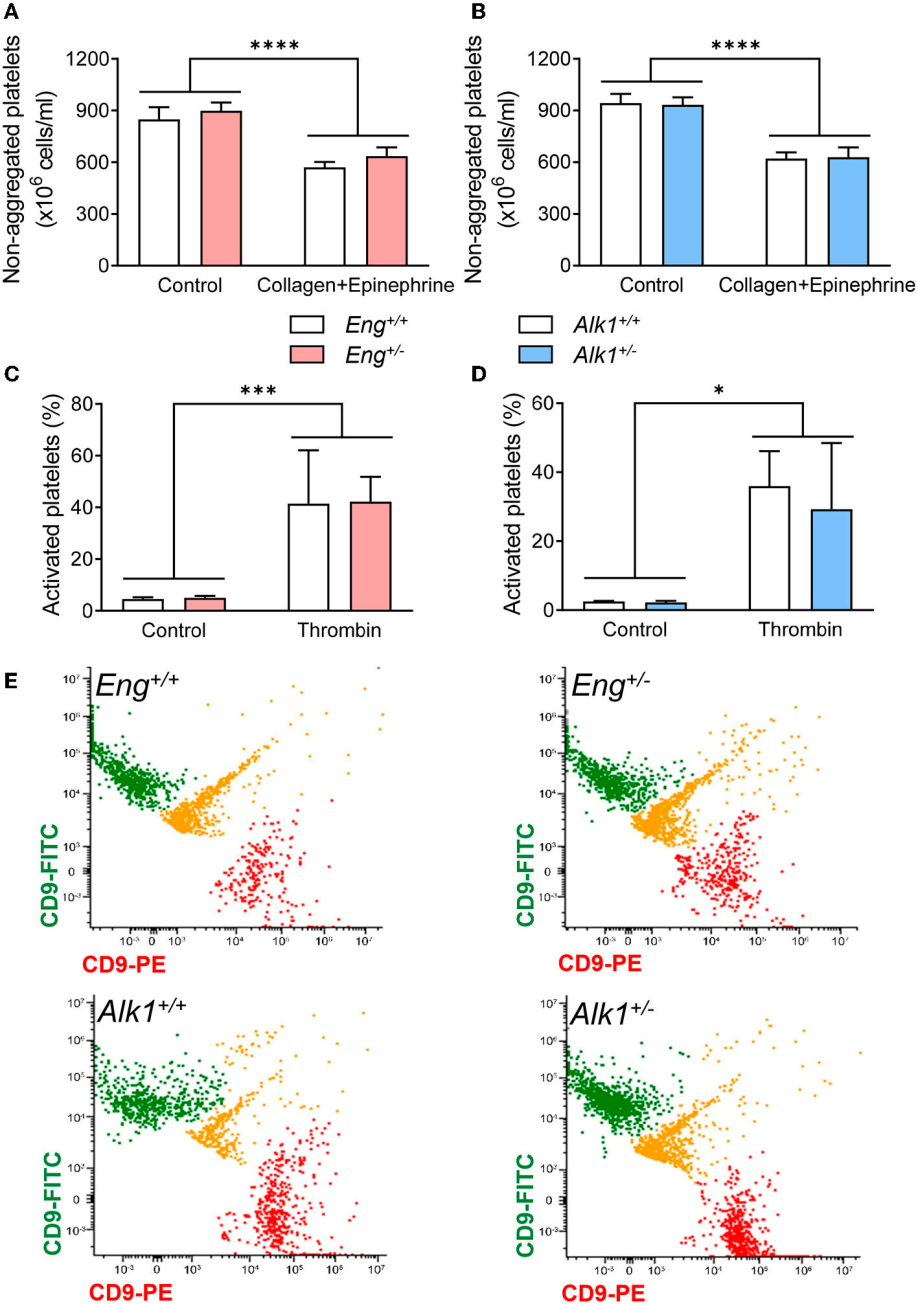
**(A,B)** Number of non-aggregated platelets per mL before and after the induction of a thrombogenic event with a bolus of collagen and epinephrine in the HHT-1 murine model **(A)**, *Eng*^+/−^ mice (*n* = 8) and their respective controls *Eng*^+/+^ (*n* = 9); and in the HHT-2 murine model **(B)**, *Alk1*^+/−^ mice (*n* = 10) and their respective controls *Alk1*^+/+^ (*n* = 14). **(C,D)** Percentage of activated platelets, determined by flow cytometry, after treatment with thrombin of diluted blood from *Eng*^+/−^ mice (*n* = 7 mice) and their respective controls *Eng*^+/+^ (*n* = 6 mice) **(C)** or from *Alk1*^+/−^ mice (*n* = 6 mice) and their controls *Alk1*^+/+^ (*n* = 5 mice) **(D)**. **(E)** Representative images, from 3 independent experiments, of flow cytometry analysis of platelet aggregation after thrombin stimulation of diluted blood from *Eng*^+/+^, *Eng*^+/−^, *Alk1*^+/+^, and *Alk1*^+/−^. Platelets are divided into 2 populations that are labeled with PE or with FITC bound in both cases to CD9. If the thrombin stimulus stimulates aggregation, green and red colocalization events are observed, labeled in yellow. Results were considered statistically significant if *p* < 0.05 (ns: non-significant; *: *p* < 0.05; **: *p* < 0.01; ***: *p* < 0.001; ****: *p* < 0.0001).

Primary hemostasis depends on the ability of platelets to activate and aggregate in response to different stimuli. For *in vivo* analysis of these processes we studied the effect of intravenous injection of a sublethal mixture of collagen with epinephrine, which causes the activation and aggregation of circulating platelets forming thrombi throughout the vessels. Thus, after treatment, a decrease in non-aggregated platelets is observed, which correspond to those platelets that have formed aggregates. We found a significant difference between control and collagen/epinephrime treatment but no significant differences between *WT* and *Eng*^+/−^ or *Alk1*^+/−^ mice (Figures 3A,B). Thus, our results suggest that Eng or Alk1 deficiency does not alter the ability of platelets to activate or form platelet aggregates.

We confirmed these results by *in vitro* experiments performed with platelets isolated from *Eng*^+/−^ or *Alk1*^+/−^ mice. As shown in Figure 3, platelets from both mice are able to activate (Figures 3C,D) or aggregate (Figures 3E,F) in response to thrombin, with no differences in these processes between Eng- or Alk1-deficient mice and their respective controls.

Because the excessive bleeding in *Eng*^+/−^ and *Alk1*^+/−^ mice does not appear to be due to a defect in coagulation, a defective contractile response or defects in platelet function, we posit that HHT proteins, predominantly expressed in endothelial cells, could be involved in later stage endothelium-dependent processes of hemostasis, namely thrombus stabilization or fibrinolysis.

### Endoglin Deficiency Impairs Platelet-Endothelium Adhesion and Thrombus Stabilization

**Figure 4:**
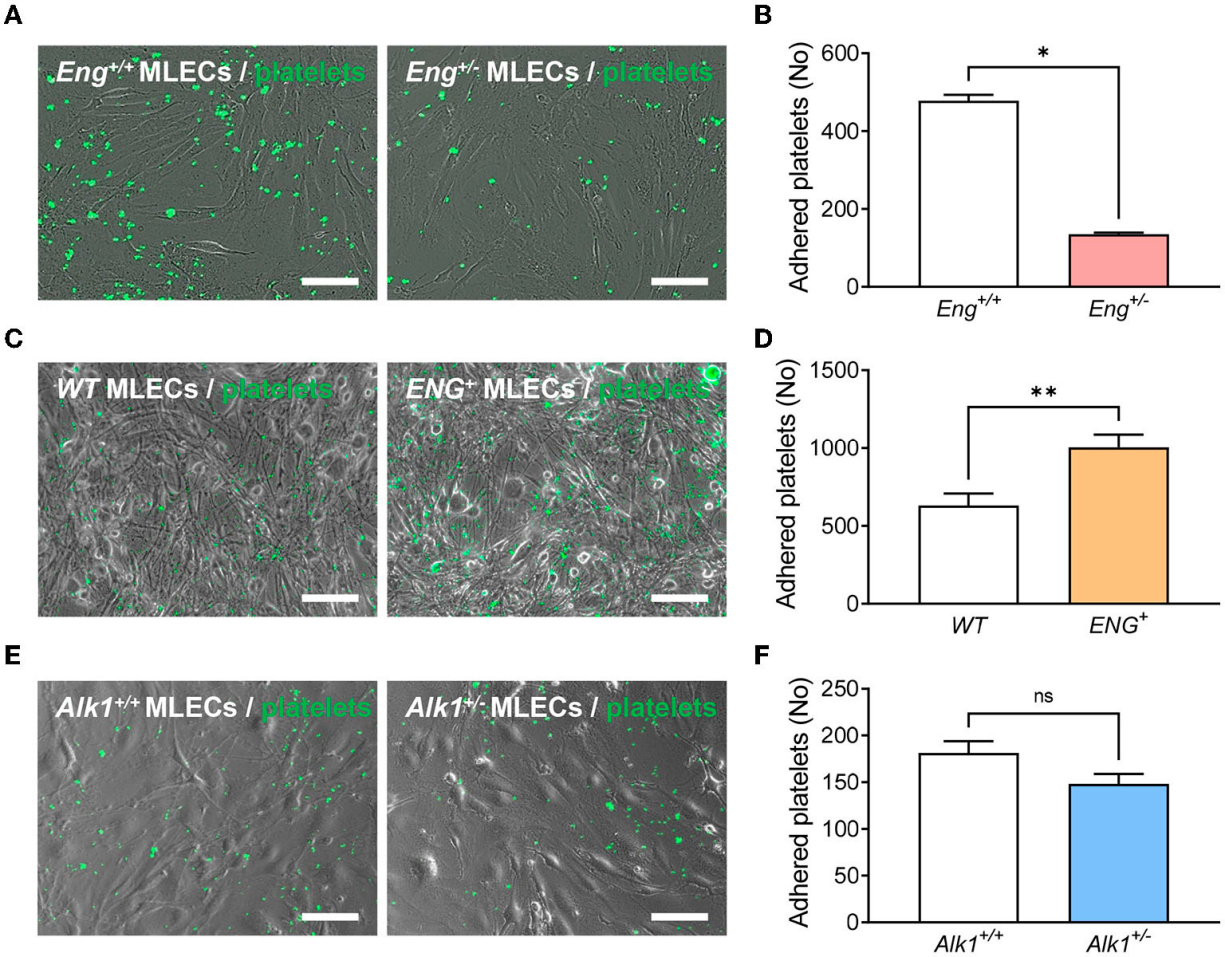
**(A)** Representative images of calcein-labeled platelets (green fluorescence) adhered on a monolayer of endothelial cells (phase contrast image), isolated from *Eng*^+/+^ or *Eng*^+/−^ mice, treated with CXCL12. **(B)** Number of adherent platelets after 30 min of incubation of platelets on CXCL12-activated endothelial cells isolated from *Eng*^+/+^ or *Eng*^+/−^ mice (*n* = 4). **(C)** Representative images of calcein-labeled platelets (green fluorescence) adhered on a monolayer of endothelial cells (phase contrast image), isolated from *WT* or *ENG*^+^ mice, treated with CXCL12. **(D)** Number of adherent platelets after 30 min of incubation of platelets on CXCL12-activated endothelial cells isolated from *WT* or *ENG*^+^ mice (*n* = 9). **(E)** Representative images of calcein-labeled platelets (green fluorescence) adhered on a monolayer of endothelial cells (phase contrast image), isolated from *Alk1*^+/+^ or *Alk1*^+/−^ mice, treated with CXCL12. **(F)** Number of adherent platelets after 30 min of incubation of platelets on CXCL12-activated endothelial cells isolated from *Alk1*^+/+^ or *Alk1*^+/−^ mice (*n* = 5). Results were considered statistically significant if *p* < 0.05 (ns: non-significant; *: *p* < 0.05; **: *p* < 0.01; ***: *p* < 0.001; ****: *p* < 0.0001).

Although the quiescent endothelium is remarkably refractory to platelet adhesion, platelet-endothelium adhesion can occur after endothelial stimulation and this mechanism is important for proper thrombus stabilization. Recently, we have described that endoglin may participate in this interaction between platelets and endothelium through integrin binding (17). We therefore analyzed whether Eng, or Alk1, heterozygosity in the endothelium was causing reduced platelet adhesion. To this end, monolayers of MLECs from *Eng*^+/−^ and *Alk1*^+/−^ animals and their respective control mice, were incubated for 30 min with calcein-labeled platelets and the integrin activator CXCL12 and binding of platelets was measured. We found that platelets displayed a lower adhesion to *Eng*^+/−^ MLECs compared to controls (*Eng*^+/+^ MLECs) (Figures 4A,B). In addition, to confirm that the effect is dependent on endoglin expression, we analyzed platelet adhesion to MLECs from *ENG*^+^ mice, that constitutively and ubiquitously express high levels of endoglin. Our results show that adhesion to ENG^+^ MLECs was significantly higher than to WT MLECs (Figures 4C,D). Interestingly, this result does not hold in Alk1-deficient cells, since no significant differences were found when we compared platelet adhesion to *Alk1*^+/−^ vs. *Alk1*^+/+^ MLECs (Figures 4E,F).

Next, we assessed the role of Eng in resistance to shear under flow, using microfluidic devices. Platelets were allowed to adhere to endothelial cells, previously stimulated for 10 min with the integrin activator CXCL12, and the resistance to perfusion at 2 dynes/cm^2^ for 2 min was measured (Supplementary Figures S3A,B, Supplementary Videos 1, 2). We found that platelets resistance to flow was strongly reduced in *Eng*^+/−^ compared to *WT* endothelial monolayers. Similar to the static adhesion results, the resistance to shear flow of platelets attached to the *Alk1*^+/−^ MLECs monolayer is comparable to that of *WT* MLECs (Supplementary Figures S3C,D and Supplementary Videos 3, 4). These results suggest that the presence of Eng in the monolayer of MLECs contributes to guarantee a platelets' firm adhesion and this may be critical to ensure proper thrombus formation and stabilization.

**Figure 5:**
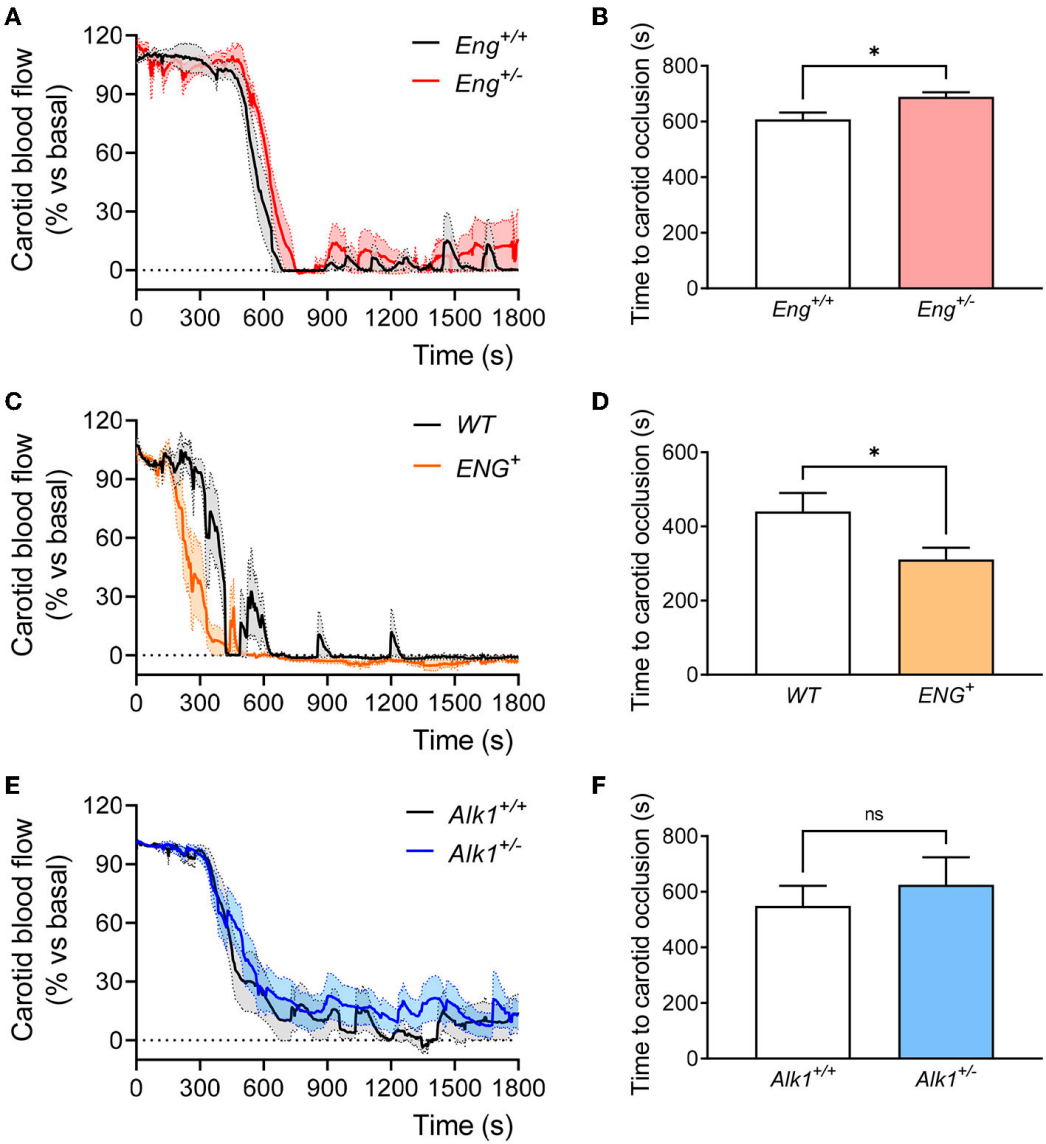
**(A)** Evolution of carotid blood flow after application of FeCl_3_ in carotid arteries of *Eng*^+/+^ (*n* = 5) and *Eng*^+/−^ (*n* = 5) mice. Percentage of baseline blood flow is represented as the mean of all mice (thick line) and the standard error of the mean (dotted line and shaded area). **(B)** Time to complete a stable carotid occlusion after FeCl_3_ administration in carotids of *Eng*^+/−^ mice (*n* = 5) and their controls (*n* = 5). **(C)** Evolution of carotid blood flow after application of FeCl_3_ in carotid arteries of *WT* (*n* = 5) and *ENG*^+^ (*n* = 6) mice. Percentage of baseline blood flow is represented as the mean of all mice (thick line) and the standard error of the mean (dotted line and shaded area). **(D)** Time to complete a stable carotid occlusion after FeCl_3_ administration in carotids of *ENG*^+^ mice (*n* = 6) and their *WT* controls (*n* = 5). **(E)** Evolution of carotid blood flow after application of FeCl_3_ in carotid arteries of *Alk1*^+/+^ (*n* = 10) and *Alk1*^+/−^ (*n* = 12) mice. Percentage of baseline blood flow is represented as the mean of all mice (thick line) and the standard error of the mean (dotted line and shaded area). **(F)** Time to complete a stable carotid occlusion after FeCl_3_ administration in carotids of *Alk1*^+/−^ mice (*n* = 12) and their controls (*n* = 10). Results were considered statistically significant if *p* < 0.05 (ns: non-significant; *: *p* < 0.05; **: *p* < 0.01; ***: *p* < 0.001; ****: *p* < 0.0001).

Next, we analyzed thrombus formation and stabilization by measuring blood flow in the carotid artery after FeCl_3_ administration on the external carotid wall. FeCl_3_ diffuses to the endothelial layer and generates a damage capable of activating the hemostasis response and generating a thrombus that reduces and even blocks blood flow. Our results show that stable occlusion, determined as the reduction of carotid blood flow to <5% of baseline blood flow, was delayed in *Eng*^+/−^ compared to *WT* mice (Figures 5A,B). This is in agreement with the above results on platelet adhesion to the endothelium. In order to further assess the role of endoglin in these hemostasis functions, we analyzed thrombus formation in the mouse model overexpressing human endoglin (*ENG*^+^). We found that wound closure was significantly faster in *ENG*^+^ mice, which present constitutively increased levels of endoglin, than in control animals (Figures 5C,D), suggesting that endoglin is directly related to the formation of a stable thrombus and positively contributes to hemostasis. To rule out that this effect on stable thrombus formation was not due to endoglin overexpression leading to changes in platelet activity, we analyzed *in vivo* platelet activation and aggregation by measuring (i) non-aggregated platelets after the induction of a thrombogenic event in *ENG*^+^ mice and (ii) *in vitro* platelet aggregation in response to thrombin. Our results show that endoglin overexpression did not alter platelet activation or aggregation either *in vivo* (Supplementary Figure S4A) or *in vitro* (Supplementary Figures S4B,C). In addition, we analyzed the effect of endoglin overexpression on the bleeding time of these mice and also observed no difference between *ENG*^+^ and control mice (Supplementary Figure S4D).

At variance with the *Eng*^+/−^ mice, no significant differences between *Alk1*^+/−^ mice and controls were observed regarding thrombus formation and carotid closure (Figures 5E,F). This is consistent with the absence of differences in platelet adhesion to endothelial cells when comparing *Alk1*^+/−^ vs. *Alk1*^+/+^ MLECs (Figures 4E,F and Supplementary Figures S3C,D).

These results suggest that endoglin haploinsufficiency, but not Alk1 haploinsufficiency, leads to unstable thrombus formation that may explain the occurrence of rebleeding, whereas in the Alk1-deficient condition, the difficulty in stopping bleedings seems to be due to a different mechanism.

### Alk1 Deficiency Stimulates Thrombus Fibrinolysis

Fibrinolysis is involved in the process of removing the clot formed upon hemostasis activation. We analyzed the main components of the fibrinolysis cascade in plasma samples from *Eng*^+/−^, *Alk1*^+/−^ and their control mice, collected immediately before (basal levels) and 30 min, 6 or 24 h after an acute thrombotic event: the intravenous administration of collagen and epinephrine as above described. These collection times were selected by choosing the periods where the highest concentrations of each of the components are expected to be found.

**Figure 6:**
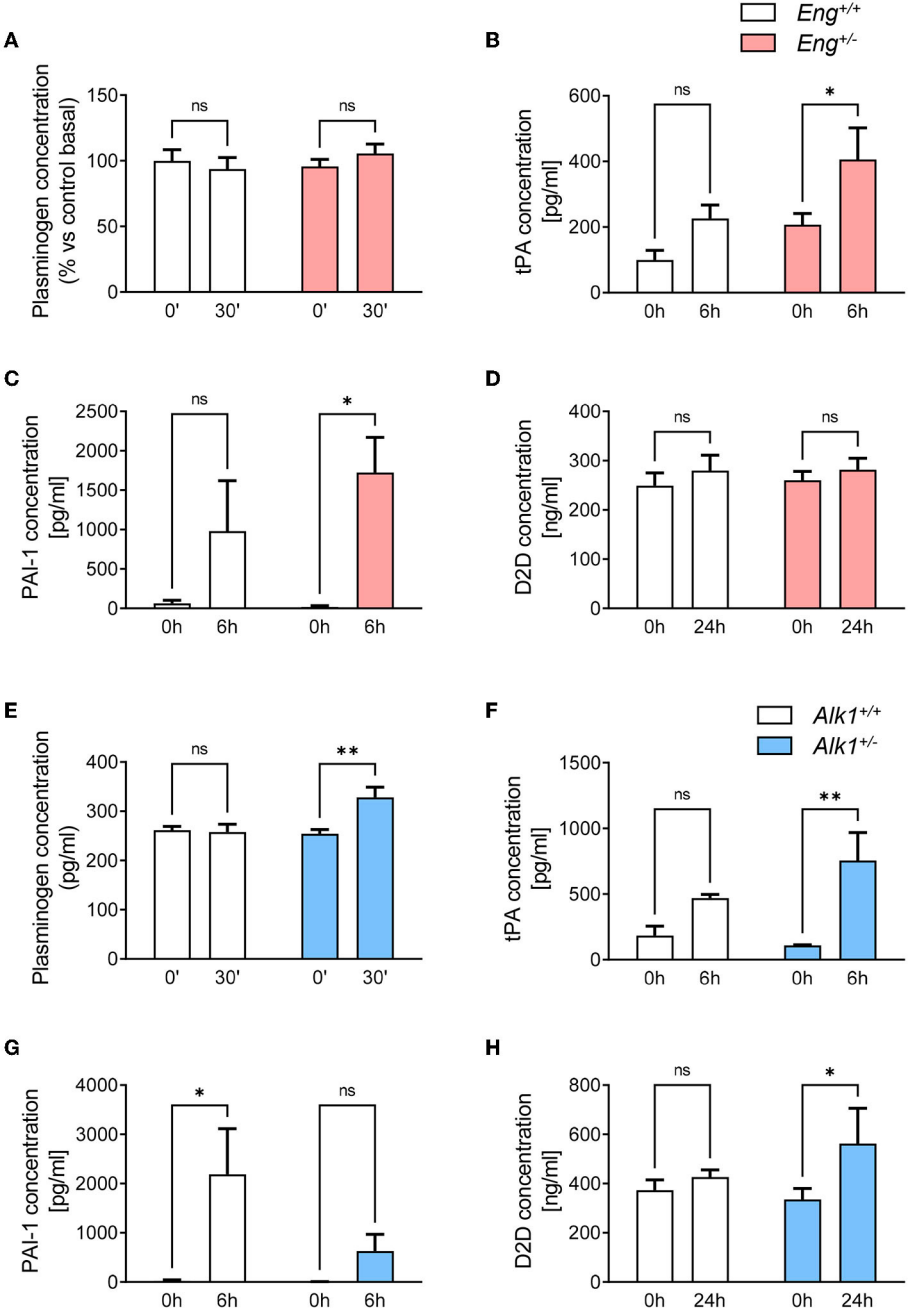
**(A)** Plasma levels of plasminogen at baseline and 30 min after a thrombogenic event in *Eng*^+/+^ (*n* = 5) and *Eng*^+/−^ (*n* = 5) mice. **(B)** Plasma levels of t-PA at baseline and 6 h after a thrombogenic event in *Eng*^+/+^ (*n* = 5) and *Eng*^+/−^ (*n* = 5) mice. **(C)** Plasma levels of PAI-1 at baseline and 6 h after a thrombogenic event in *Eng*^+/+^ (*n* = 5) and *Eng*^+/−^ (*n* = 5) mice. **(D)** Plasma levels of D-dimer at baseline and 24 h after a thrombogenic event in *Eng*^+/+^ (*n* = 5) and *Eng*^+/−^ (*n* = 5) mice. **(E)** Plasma levels of plasminogen at baseline and 30 min after a thrombogenic event in *Alk1*^+/+^ (*n* = 5) and *Alk1*^+/−^ (*n* = 5) mice. **(F)** Plasma levels of t-PA at baseline and 6 h after a thrombogenic event in *Alk1*^+/+^ (*n* = 5) and *Alk1*^+/−^ (*n* = 5) mice. **(G)** Plasma levels of PAI-1 at baseline and 6 h after a thrombogenic event in *Alk1*^+/+^ (*n* = 5) and *Alk1*^+/−^ (*n* = 5) mice. **(H)** Plasma levels of D-dimer at baseline and 24 h after a thrombogenic event in *Alk1*^+/+^ (*n* = 5) and *Alk1*^+/−^ (*n* = 5) mice. Results were considered statistically significant if *p* < 0.05 (ns: non-significant; *: *p* < 0.05; **: *p* < 0.01; ***: *p* < 0.001; ****: *p* < 0.0001).

The basal plasminogen concentration in plasma was similar in *Eng*^+/−^ and *WT* mice, and no significant changes were observed in both groups 30 min after the induction of the thrombotic event (Figure 6A). However, 6 h after the thrombotic event, tissue plasminogen activator (t-PA) (Figure 6B) and its inhibitor PAI-1 (Figure 6C) levels significantly increased in *Eng*^+/−^ mice while in the *WT* this increment is lower and not statistically significant. Finally, we analyzed the plasmatic levels of D-dimer, a breakdown product of the blood clot fibrinolysis. Our results show that 24 h after the thrombotic event, D-dimer levels do not increase significantly in either *WT* or *Eng*^+/−^mice (Figure 6D). Therefore, Eng deficiency causes a greater release not only of t-PA, but also of its inhibitor PAI-1 after the occurrence of a thrombotic event; these changes being associated with no differences in in the plasma levels of D-dimer.

Interestingly, the fibrinolytic response of the HHT-2 animal model was substantially different from that of HHT-1 mice. Thus, after the thrombotic event induced in *Alk1*^+/−^ mice, a significant increase in plasminogen levels at 30 min was observed, whereas the control group remained unresponsive (Figure 6E). Similar to the HHT-1 model, 6 h after activating thrombogenesis, there was a statistically significant increase in plasma levels of t-PA in *Alk1*^+/−^ mice, but not in *WT* mice (Figure 6F). However, contrary to what was observed in Eng-deficient mice, in *Alk1*^+/−^ mice the increase in PAI-1 levels did not reach statistical significance and was clearly much lower than the significant increase that occurred in control mice (Figure 6G). Analysis of D-dimer levels 24 h after the thrombotic event showed a significant increase in plasma levels of *Alk1*^+/−^ mice with no statistical differences in control mice (Figure 6H). In summary, the generation of a thrombotic event in the murine model of HHT-2 results in a significant increase in plasminogen and t-PA. However, the increment in PAI-1 levels is much lower than in controls. Finally, an increase in D-dimer levels is detected that is not observed in controls. Together, these results support the existence of a greater fibrinolysis activity in *Alk1*^+/−^ mice.

## Discussion

The patterns of presentation of HHT are highly variable, but spontaneous and recurrent nosebleeds is the most common clinical manifestation, affecting more than 90% of patients, and is usually the first to appear; thus, it is one of the established Curaçao Criteria for the diagnosis of HHT. In addition, over the progression of the disease, 15–20% of individuals eventually develop recurrent bleeding from the gastrointestinal tract (36). Nosebleeds, GI bleeds, or both, cause a large proportion of HHT patients to suffer chronic anemia, and the need to receive periodic blood transfusions in those patients unresponsive to iron supplementation treatments (37).

Currently it is widely considered that the increased bleeding of HHT patients is due to the abnormalities and fragility of the vasculature (35, 38). For this reason, most of HHT-related studies have focused on angiogenesis and the defective structure of telangiectases. However, whereas the fragility of telangiectases could explain the higher frequency of hemorrhages, it is unclear why bleeds are so intense, especially considering that other pathologies that also present telangiectases, lack the intense bleeding characteristic of HHT (39–44). It should be noted that most other pathologies which present telangiectases rarely display them in nasal and GI mucosa, as skin telangiectases are more usually frequent, which may explain this difference. However, also among HHT patients they show a wide variation in bleeding severity, which is not completely explained by the well-known factors, such as number and size of telangiectases. Therefore, one can assume that, in addition to the fragility of the vascular lesions, an alteration in hemostasis could also participate in the intense bleeding observed in HHT patients.

Some authors have already proposed that there may be alterations in phases of primary and secondary hemostasis associated with HHT even increasing the risk of thrombosis, but most of them are old studies and there is considerable controversy among them (5, 45–50). In this regard, our results demonstrate that the presence of increased bleeding in HHT patients is not associated with lengthened clotting times or lower clotting factor activity, as suggested in the classical description of the disease (35). In that context, to elucidate if the abnormally increased bleeding intensity of HHT patients may be due to an alteration of hemostasis in non-coagulation related phases, here we have analyzed in detail the hemostasis system in two murine models of the disease. We have used mice that are haploinsufficient for the endoglin gene (*Eng*^+/−^) or for the Alk1 gene (*Alk1*^+/−^). These mice are the closest experimental models of HHT-1 and HHT-2 patients in terms of genotype (19). Of note, the HHT phenotype of these animals is highly dependent on the mouse strain. Thus, *Eng*^+/−^ and *Alk1*^+/−^ animals with 129/Ola background develop highly penetrant and frequent clinical manifestations similar to those of HHT, such as dilated vessels, nosebleeds, telangiectases and vascular lesions in the liver, the nail bed, ears, intestine and skin (24, 51, 52). By contrast, in *Eng*^+/−^ and *Alk1*^+/−^ animals with a C57BL/6J background the penetrance of the HHT phenotype is incomplete and its frequency is negligible (24, 51). Therefore, *Eng*^+/−^ and *Alk1*^+/−^ animals in the C57BL/6J background, as used in this work, represent a useful model to analyze the hemostasis system in the absence of vascular lesions that could affect the bleeding. Regardless, the findings obtained using the C57BL/6J genetic background should be confirmed in future studies using other genetic backgrounds.

When analyzing in the HHT animal models the bleeding from a normal vessel, that does not involve telangiectases or arteriovenous malformations, we observe that both murine models (*Eng*^+/−^ and *Alk1*^+/−^) have more abundant rebleeding than controls (Figure 2). These results suggest an alteration in the mechanisms of hemostasis that may account for the difficulty in stopping the bleeding in HHT patients, prompting us to analyze the different phases involved in hemostasis, e.g., vessel spasm, formation of platelet plug, thrombus stabilization and thrombus dissolution (fibrinolysis).

Previous studies have described the role of endoglin or ALK1 in vascular tone, where the endothelium has a main role in the secretion of vasoactive molecules (53). Thus, *Eng*^+/−^ mice present reduced nitric oxide-dependent vasodilation (27) and *Alk1*^+/−^ mice show increased arterial pressure due to sympathetic activation (54). Moreover, heterozygous mutations in either *ENG* or *ALK1* in humans are associated, in a few cases, with pulmonary arterial hypertension due their functional link with the BMP type II receptor, the main target responsible for most family cases of pulmonary arterial hypertension (46, 55). Because these findings suggest that a deficient expression of endoglin or ALK1 contributes to the deterioration of vascular tone, we analyzed whether the vasoconstrictor response of aortic rings was impaired as a consequence of the genetic alteration in *Eng*^+/−^ and *Alk1*^+/−^ mice. Our results, using three vasoactive agents present in the hemostatic response, only exhibited an increased contraction in response to serotonin in the aortic rings from *Eng*^+/−^ mice when compared with the control, whereas no significant impairment in the contractile response of aortic rings from the two HHT mouse models with respect to their controls was observed. These results support that the greater bleeding of *Eng*^+/−^ and *Alk1*^+/−^ mice is not due to less vascular spasm. They also reinforce the idea that the effect of these genes on the regulation of vascular tone is primarily due to alterations in vasodilation rather than vascular contraction, although it might be interesting to investigate the outcome in response to serotonin in endoglin-deficient mice in case it contributes to the relationship between endoglin and pulmonary hypertension.

With regard to thrombus formation, we show that, compared to control animals, *Eng*^+/−^ mice take longer time to occlude the carotid artery after FeCl_3_-induced local thrombotic response. This effect does not seem to depend on the ability of platelets to activate and form aggregates, since no differences in these processes are observed between *Eng*^+/−^ mice and controls. Therefore, as previously hypothesized (17), *Eng* deficiency seems to be involved in thrombus instability. This is corroborated by platelet adhesion assays on monolayers of *Eng*^+/+^ and *Eng*^+/−^ MLECs, which show that Eng deficiency in endothelial cells compromises platelet adhesion and their resistance to shear stress, whereas endoglin-overexpressing MLECs show increased platelet adhesion to these endothelial cells, supporting the adhesive role of endoglin in the interaction between platelets and activated endothelium, as previously proposed (17). This conclusion is reflected in the fact that mice with overexpression of endoglin are able to generate a thrombus stable enough to occlude the carotid in a shorter time than *WT* mice. Interestingly, this does not translate into a shorter bleeding time, so it seems that the physiological levels of Eng are sufficient to ensure a correct hemostasis.

Surprisingly, a different situation was found in *Alk1*^+/−^ mice, the murine model for HHT-2, since, in this case the excessive bleeding does not appear to be due to alterations in platelet-endothelium adhesion. Similarly to *Eng*-deficient mice, the rebleeds of *Alk1*-deficient mice are more severe than those in controls, but in this case, in addition to finding no differences in platelet activation or aggregation, we also found no differences in platelet adhesion to activated endothelium or carotid occlusion time after FeCl_3_ application. Interestingly, when analyzing the fibrinolysis system, we found that the stimulation of a thrombotic event in *Alk1*^+/−^ mice result in higher plasma levels of plasminogen and its activator t-PA, and lower levels of PAI-1, the inhibitor of t-PA. These results suggest that Alk1 deficiency may result in an increased fibrinolysis and higher degradation of the thrombus that may account for the increased levels of D-Dimer found in these animals and severe rebleedings. Of note, a relationship between ALK1 deficiency and increased t-PA has been previously reported (25). Thus, in line with our results, abnormally high levels of t-PA have been shown in *Alk1* knock-out mice (25) and in telangiectases of HHT patients (56, 57). However, although there is sufficient evidence that ALK1 deficiency leads to an increase in PAI-1 (25, 58), our results point in the opposite direction. The studies that relate ALK1 deficiency with PAI-1 are studies on TGFβ1 signaling pathways in which 2 pathways are described with opposite effects depending on whether it signals through ALK1 or ALK5, both TGFβ1 type I receptors. Thus, if there is ALK1 deficiency, signaling would be mainly through ALK5, giving rise to different cellular responses. In this context, PAI-1 has traditionally been used as a reporter of the activity of the TGFβ1-ALK5 pathway, so it seems that ALK1 deficiency would have to cause an increase in PAI-1. However, these studies did not analyze the accumulation of PAI-1 in plasma but its cellular expression in response to treatments and therefore we consider that our results do not contradict the literature but may be a consequence of the excessively high levels of t-PA. The way PAI-1 inhibits t-PA is by forming covalent bonds that are rapidly degraded (59, 60). Thus, if t-PA levels are very high, as they are in ALK1-deficient mice, the rise in PAI-1 would be masked by its increased binding to t-PA and its rapid elimination.

**Figure 7:**
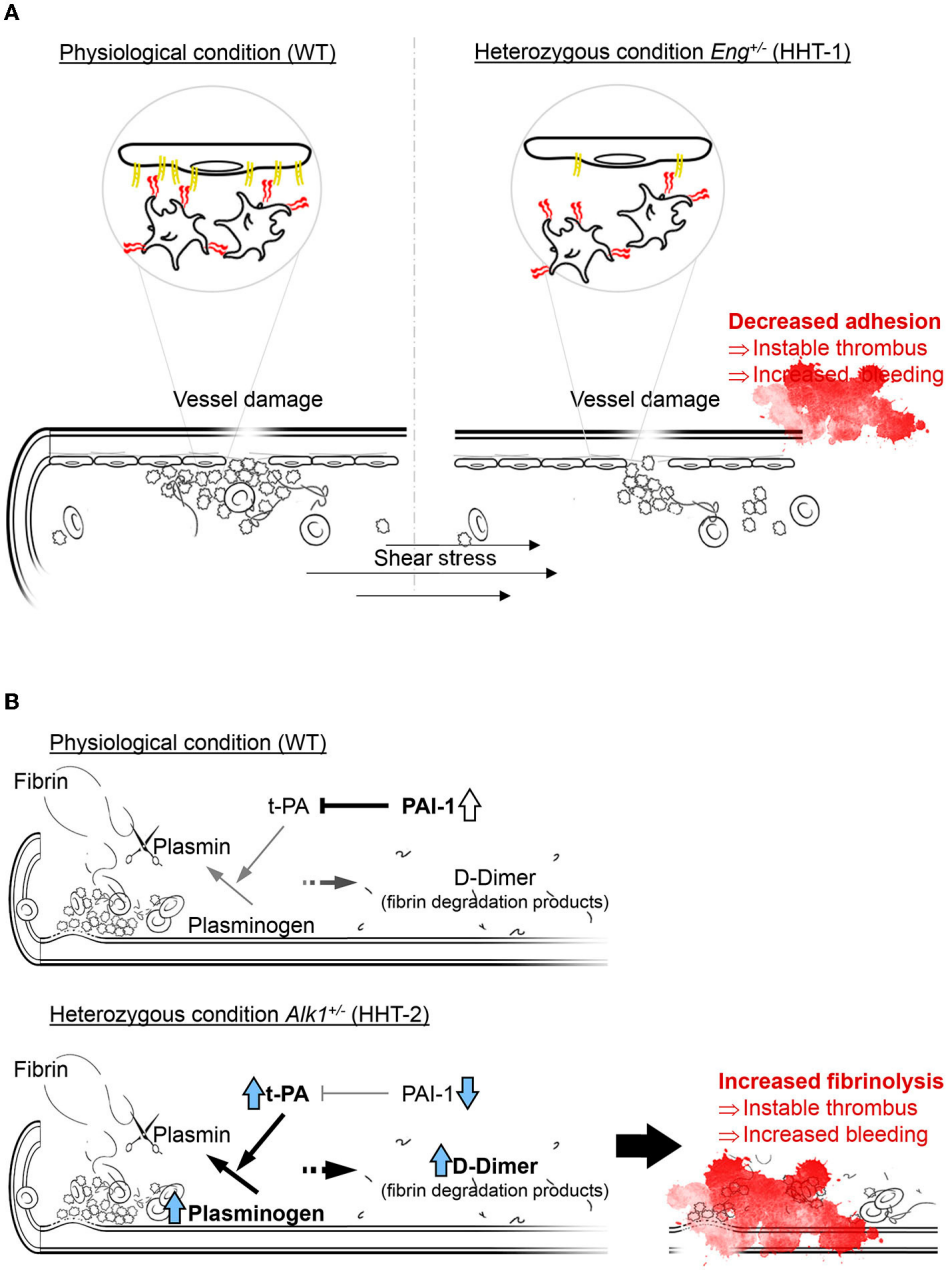
**(A)** In normal conditions platelets rolling on endothelial cells is effective and the thrombus generated is stable, while a loss on endoglin in the endothelium mean less effective platelets adhesion and an instable thrombus that can be carried away by the bloodstream, compromising thrombosis and increasing bleeding. Thus, endoglin-deficient mice need more time to generate a thrombus large and stable enough not to be entrained. **(B)** Fibrinolysis depends on the activity of t-PA catalyzing the conversion of plasminogen to plasmin, which is the enzyme responsible for fibrin degradation and thrombus disruption. In physiological conditions, there is an increase in PAI-1 that inhibits the function of t-PA and allows stabilization of the newly formed thrombus. However, ALK1 deficiency causes plasminogen and mainly t-PA levels to be much higher, so that PAI-1 cannot inhibit it and fibrinolysis is increased, which we can detect by the increase in D-dimer.

Taken together, our findings allow us to suggest the model presented in Figure 7. In a condition of endoglin deficiency, as in HHT-1 patients or *Eng*^+/−^ mice, platelets interact with each other and with the sub-endothelium to generate an apparently correct platelet plug. However, the deficient expression of endoglin in the endothelium results in suboptimal platelet-endothelium interactions, impairing the formation of a robust and stable thrombus. This unstable thrombus could be torn off and dragged by the mechanical force exerted by the bloodstream, which would result in the reappearance of the hemorrhage (Figure 7A). After the onset of a thrombogenic event, the fibrinolysis cascade is set in motion, which allows fibrin to be degraded by activation of plasminogen to plasmin by t-PA. Under physiological conditions a significant increase in the t-PA inhibitor, PAI-1, prevents excessive fibrinolysis that could compromise the integrity of the newly formed thrombus. However, ALK1 deficiency results in a much greater increase in t-PA that PAI-1 is unable to effectively control. This results in increased fibrin degradation leading to instability of the newly formed thrombus and increased risk of rebleeding (Figure 7B).

In summary, our results support the hypothesis that in HHT-1 and HHT-2 mouse models, Eng and Alk1 haploinsufficiency leads to a common phenotype of impaired hemostasis but through two different mechanisms: Eng haploinsufficiency affects thrombus stabilization due to deficient interactions between platelets and endothelial cells, while Alk1 deficiency generates an upregulation of the fibrinolysis system. This contribution could open new therapeutic approaches not only in the management of nosebleeds and gastrointestinal bleeding in HHT patients, but also to the understanding of the effect of other endothelium- or thrombosis-related pathologies.

## Data Availability Statement

The raw data supporting the conclusions of this article will be made available by the authors, without undue reservation.

## Ethics Statement

The studies involving human participants were reviewed and approved by Comité de Ética del Complejo Asistencial Universitario de Salamanca. The patients/participants provided their written informed consent to participate in this study. The animal study was reviewed and approved by Comité de Bioética de la Universidad de Salamanca.

## Author Contributions

CE-T, ER, CO-I, MP-G, JMB, JG-P, AR-B, CB, JL-N, and MP: conceptualization. CE-T, ER, MS, JMB, JG-P, and MP: methodology. CE-T, ER, MP-G, and MP: formal analysis. CE-T, ER, CO-I, MP-G, MS, and MP: investigation. ER, MP-G, MS, JG-P, AR-B, CB, JL-N, and MP: resources. ER and MP: writing—original draft preparation. CE-T, ER, and MP: visualization. MP: supervision and project administration. ER, CB, and MP: funding acquisition. All authors: writing—review and editing. All authors have read and agreed to the published version of the manuscript.

## Funding

This work was supported by the Junta de Castilla y León (BIO/SA70/14, GRS2135/A/2020, and GRS2314/A/2021), Fundación Mutua Madrileña (FMM AP172142019), the Instituto de Salud Carlos III (PI16/00460 and PI19/01630, co-funded by FEDER), and Consejo Superior de Investigaciones Científicas (CSIC; 201920E022 to CB). CO-I was supported by a contract from the Ministerio de Economía y Competitividad of Spain. CE-T was a fellow from the Fundación Miguel Casado San José.

## Conflict of Interest

The authors declare that the research was conducted in the absence of any commercial or financial relationships that could be construed as a potential conflict of interest.

## Publisher's Note

All claims expressed in this article are solely those of the authors and do not necessarily represent those of their affiliated organizations, or those of the publisher, the editors and the reviewers. Any product that may be evaluated in this article, or claim that may be made by its manufacturer, is not guaranteed or endorsed by the publisher.

## Supplementary Material

Supplementary Figure S1 **(A–H)** Plasma activity of factors involved in coagulation cascade in controls (*n* = 10), HHT-1 (*n* = 5), and HHT-2 (*n* = 21) patients. The factors analyzed were Factor II **(A)**, Factor V **(B)**, Factor VII **(C)**, Factor VIII **(D)**, Factor IX **(E)**, Factor X **(F)**, Factor XI **(G)**, and Factor vW **(H)**. Results were considered statistically significant if *p* < 0.05 (ns: non-significant; ^*^: *p* < 0.05; ^**^: *p* < 0.01; ^***^: *p* < 0.001; ^****^: *p* < 0.0001).

Supplementary Figure S2 **(A–F)** Vascular contraction in response to increasing concentrations of vasoconstrictor agents, expressed as % of contraction compared with KPSS-induced contraction, of aortic rings isolated from HHT murine models and its controls. Mean ± SEM for each concentration and nonlin fit curve, calculated with the model: log(agonist) vs. response (three parameters) of Graphpad 9, are represented. **(A,B)** Vascular contraction in response to norepinephrine of aortic rings isolated from *Eng*^+/−^ mice (*n* = 20 aortic rings from 5 mice) and its respective controls *Eng*^+/+^ (*n* = 20 aortic rings from 5 mice) **(A)** or from *Alk1*^+/−^ mice (*n* = 10 aortic rings from 5 mice) and its controls *Alk1*^+/+^ (*n* = 10 aortic rings from 5 mice) **(B)**. **(C,D)** Vascular contraction in response to serotonin of aortic rings isolated from *Eng*^+/−^ mice (*n* = 10 aortic rings from 5 mice) and its respective controls *Eng*^+/+^ (*n* = 10 aortic rings from 5 mice) **(C)** or from *Alk1*^+/−^ mice (*n* = 12 aortic rings from 5 mice) and its controls *Alk1*^+/+^ (*n* = 12 aortic rings from 5 mice) **(D)**. **(E,F)** Vascular contraction in response to U-46619 (thromboxane analog) of aortic rings isolated from *Eng*^+/−^ mice (*n* = 10 aortic rings from 5 mice) and its respective controls *Eng*^+/+^ (*n* = 10 aortic rings from 5 mice) **(E)** or from *Alk1*^+/−^ mice (*n* = 8 aortic rings from 5 mice) and its controls *Alk1*^+/+^ (*n* = 8 aortic rings from 5 mice) **(F)**. Results were considered statistically significant if *p* < 0.05 (ns: non-significant; ^*^: *p* < 0.05; ^**^: *p* < 0.01; ^***^: *p* < 0.001; ^****^: *p* < 0.0001).

Supplementary Figure S3 **(A)** Representative images of calcein-labeled platelets (green fluorescence) adhered on a monolayer of endothelial cells, isolated from *Eng*^+/+^ or *Eng*^+/−^ mice, treated with CXCL12 before (left panel) or after (right panel) the application of a flow of 2 dyne/cm^2^ (yellow arrow: flow direction). After applying shear stress most platelets are removed, but some withstand the drag and remain attached to the endothelium (white arrowheads). **(B)** Fluorescence intensity of adhered platelets on CXCL12-activated endothelial cells isolated from *Eng*^+/+^ or *Eng*^+/−^ mice after the application of a flow of 2 dyne/cm^2^ for 2 min (*n* = 3). **(C)** Representative images of calcein-labeled platelets (green fluorescence) adhered on a monolayer of endothelial cells, isolated from *Alk1*^+/+^ or *Alk1*^+/−^ mice, treated with CXCL12 before (left panel) or after (right panel) the application of a flow of 2 dyne/cm^2^ (yellow arrow: flow direction). After applying shear stress most platelets are removed, but some withstand the drag and remain attached to the endothelium (white arrowheads). **(D)** Fluorescence intensity of adhered platelets on CXCL12-activated endothelial cells isolated from *Alk1*^+/+^ or *Alk1*^+/−^ mice after the application of a flow of 2 dyne/cm^2^ for 2 min (*n* = 3). Results were considered statistically significant if *p* < 0.05 (ns: non-significant; ^*^: *p* < 0.05; ^**^: *p* < 0.01; ^***^: *p* < 0.001; ^****^: *p* < 0.0001).

Supplementary Figure S4 **(A)** Number of non-aggregated platelets per mL before and after the induction of a thrombogenic event with a bolus of collagen and epinephrine in *ENG*^+^ mice (*n* = 17) and its respective *WT* controls (*n* = 17). **(B)** Total bleeding time, calculated as the sum of first bleeding time and rebleeding time, in *WT* (*n* = 25) and *ENG*^+^ mice (*n* = 25). **(C)** Representative images, from 3 independent experiments, of flow cytometry analysis of platelet aggregation after thrombin stimulation of diluted blood from *WT* and *ENG*^+^ mice. Results were considered statistically significant if *p* < 0.05 (ns: non-significant; ^*^: *p* < 0.05; ^**^: *p* < 0.01; ^***^: *p* < 0.001; ^****^: *p* < 0.0001).
