# Supplementary figures and images for: Functional Alterations Involved in Increased Bleeding in Hereditary Hemorrhagic Telangiectasia Mouse Models
Source: Front Med (Lausanne). 2022 May 19;9:871903. doi: 10.3389/fmed.2022.871903 (PMC9160577; doi:10.3389/fmed.2022.871903)

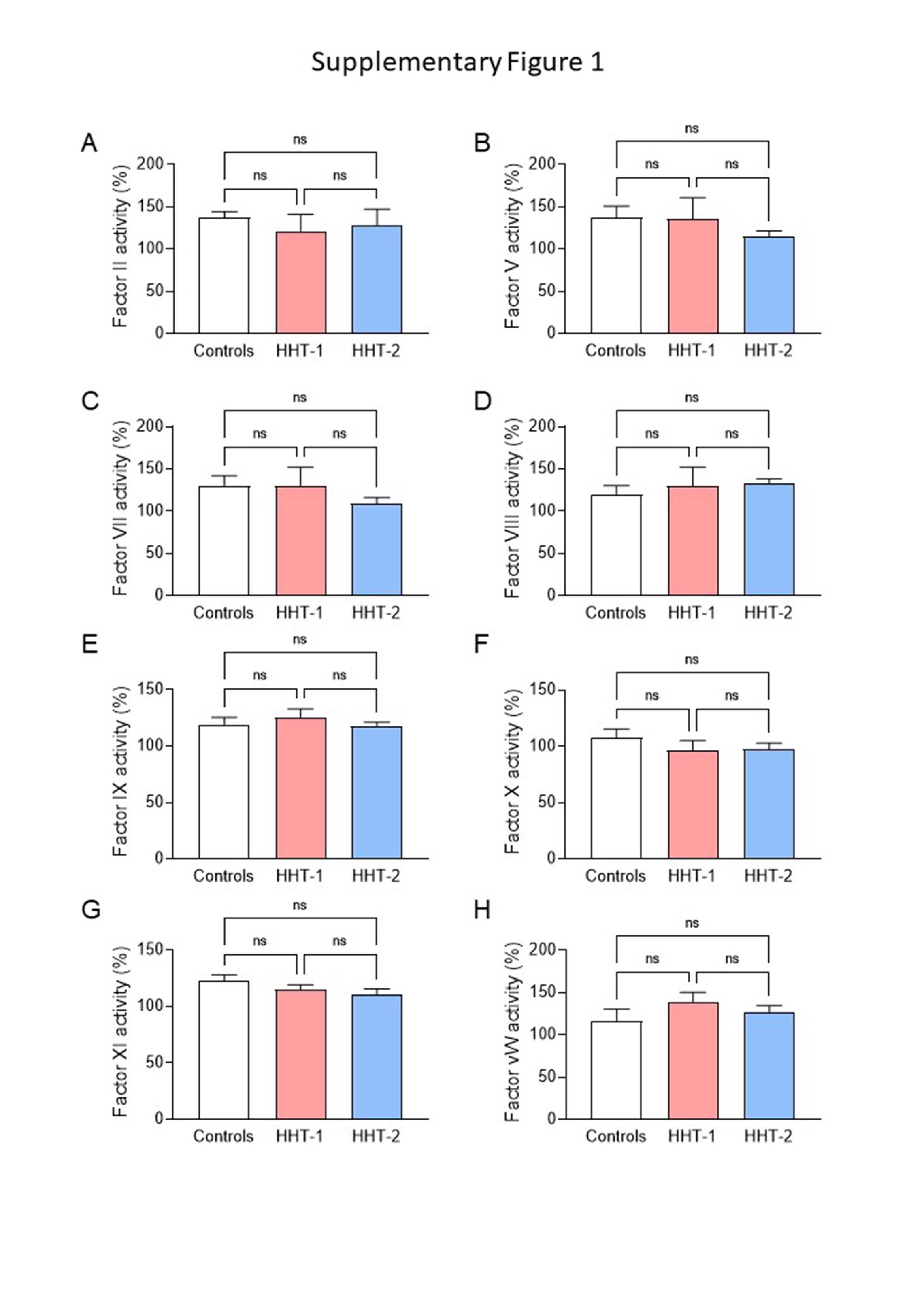

Supplement: Supplementary Figure S1 — (A–H) Plasma activity of factors involved in coagulation cascade in controls (n = 10), HHT-1 (n = 5), and HHT-2 (n = 21) patients. The factors analyzed were Factor II (A), Factor V (B), Factor VII (C), Factor VIII (D), Factor IX (E), Factor X (F), Factor XI (G), and Factor vW (H). Results were considered statistically significant if p < 0.05 (ns: non-significant; *: p < 0.05; **: p < 0.01; ***: p < 0.001; ****: p < 0.0001). [file Image_1.JPEG]

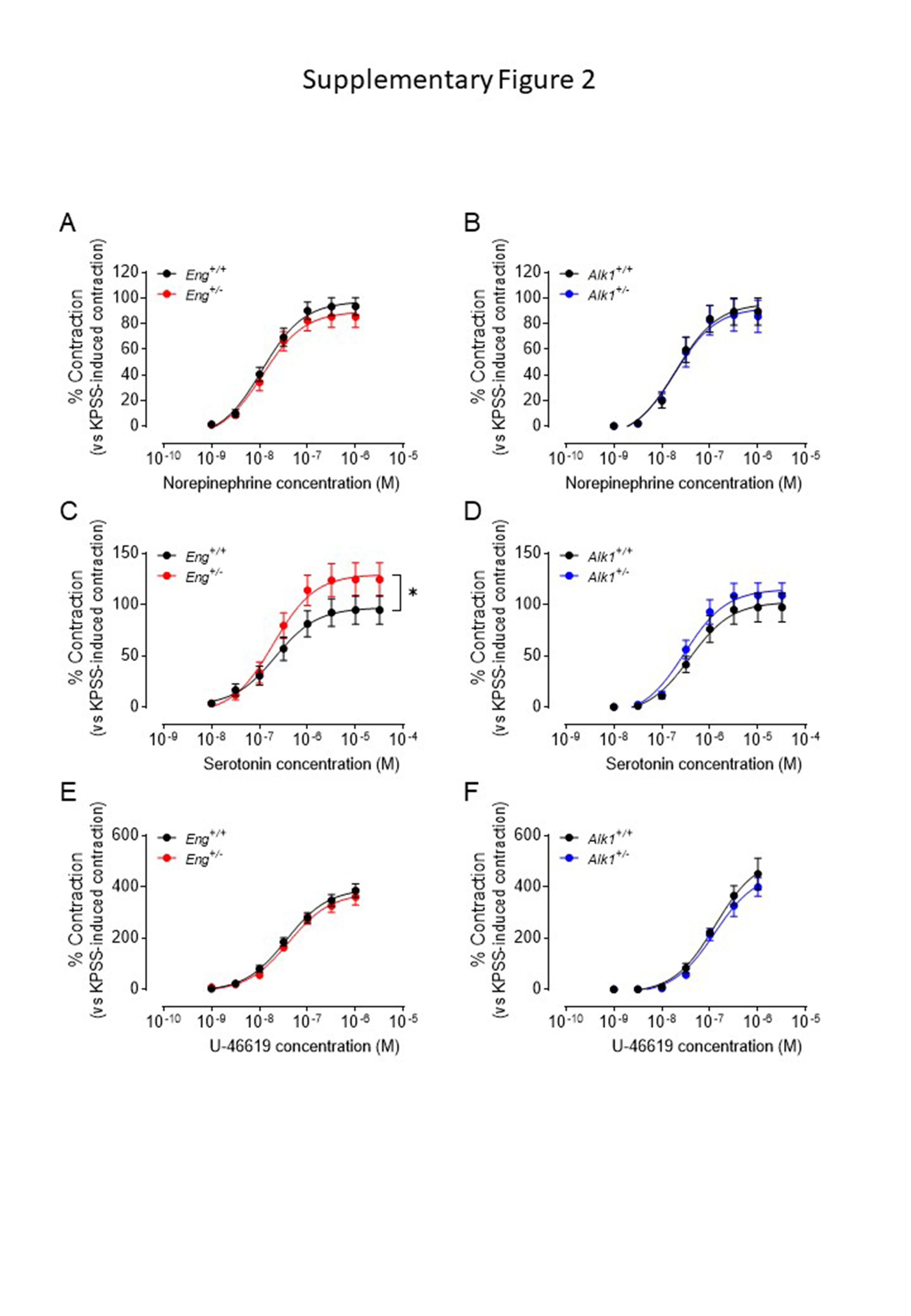

Supplement: Supplementary Figure S2 — (A–F) Vascular contraction in response to increasing concentrations of vasoconstrictor agents, expressed as % of contraction compared with KPSS-induced contraction, of aortic rings isolated from HHT murine models and its controls. Mean ± SEM for each concentration and nonlin fit curve, calculated with the model: log(agonist) vs. response (three parameters) of Graphpad 9, are represented. (A,B) Vascular contraction in response to norepinephrine of aortic rings isolated from Eng+/− mice (n = 20 aortic rings from 5 mice) and its respective controls Eng+/+ (n = 20 aortic rings from 5 mice) (A) or from Alk1+/− mice (n = 10 aortic rings from 5 mice) and its controls Alk1+/+ (n = 10 aortic rings from 5 mice) (B). (C,D) Vascular contraction in response to serotonin of aortic rings isolated from Eng+/− mice (n = 10 aortic rings from 5 mice) and its respective controls Eng+/+ (n = 10 aortic rings from 5 mice) (C) or from Alk1+/− mice (n = 12 aortic rings from 5 mice) and its controls Alk1+/+ (n = 12 aortic rings from 5 mice) (D). (E,F) Vascular contraction in response to U-46619 (thromboxane analog) of aortic rings isolated from Eng+/− mice (n = 10 aortic rings from 5 mice) and its respective controls Eng+/+ (n = 10 aortic rings from 5 mice) (E) or from Alk1+/− mice (n = 8 aortic rings from 5 mice) and its controls Alk1+/+ (n = 8 aortic rings from 5 mice) (F). Results were considered statistically significant if p < 0.05 (ns: non-significant; *: p < 0.05; **: p < 0.01; ***: p < 0.001; ****: p < 0.0001). [file Image_2.JPEG]

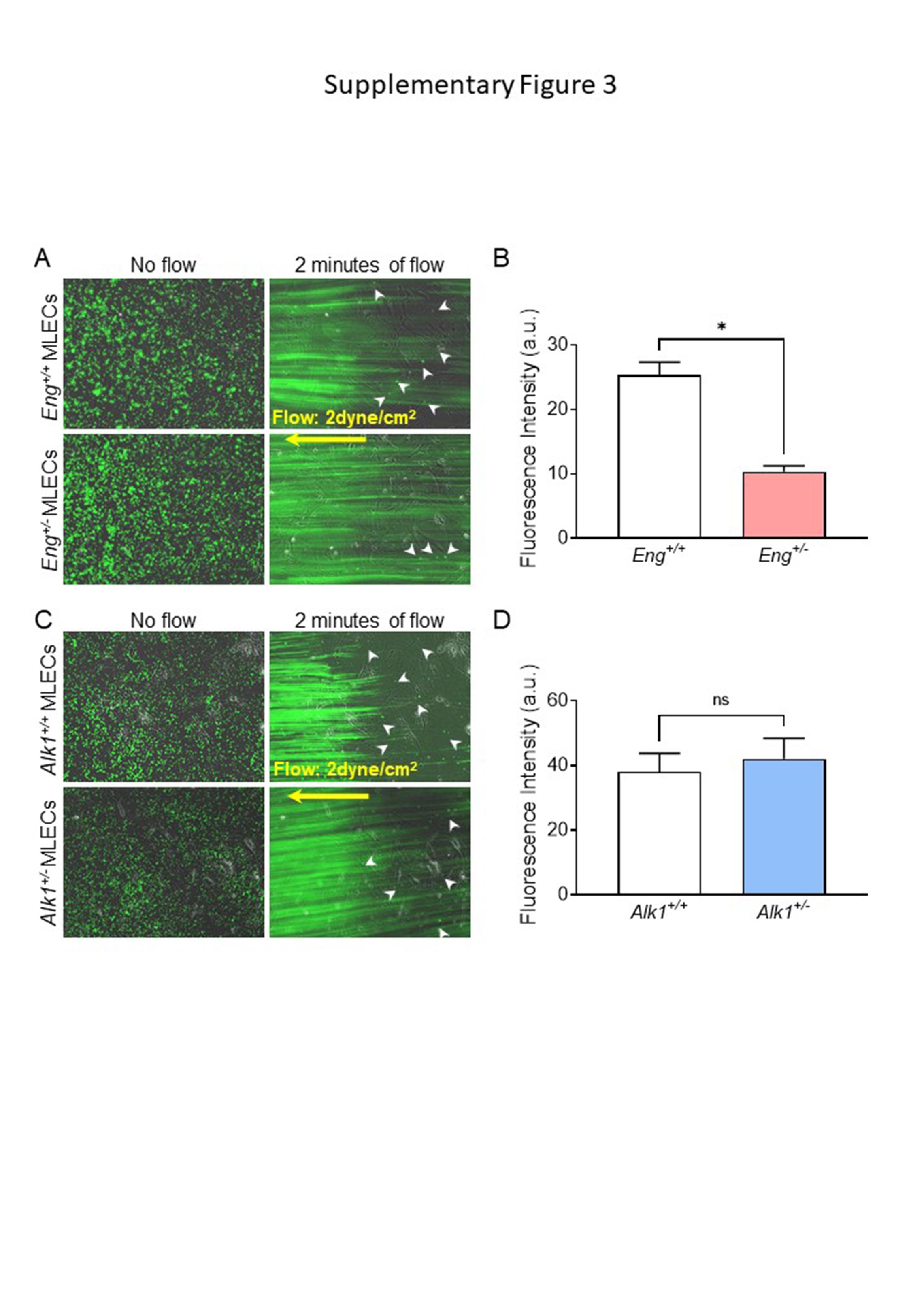

Supplement: Supplementary Figure S3 — (A) Representative images of calcein-labeled platelets (green fluorescence) adhered on a monolayer of endothelial cells, isolated from Eng+/+ or Eng+/− mice, treated with CXCL12 before (left panel) or after (right panel) the application of a flow of 2 dyne/cm2 (yellow arrow: flow direction). After applying shear stress most platelets are removed, but some withstand the drag and remain attached to the endothelium (white arrowheads). (B) Fluorescence intensity of adhered platelets on CXCL12-activated endothelial cells isolated from Eng+/+ or Eng+/− mice after the application of a flow of 2 dyne/cm2 for 2 min (n = 3). (C) Representative images of calcein-labeled platelets (green fluorescence) adhered on a monolayer of endothelial cells, isolated from Alk1+/+ or Alk1+/− mice, treated with CXCL12 before (left panel) or after (right panel) the application of a flow of 2 dyne/cm2 (yellow arrow: flow direction). After applying shear stress most platelets are removed, but some withstand the drag and remain attached to the endothelium (white arrowheads). (D) Fluorescence intensity of adhered platelets on CXCL12-activated endothelial cells isolated from Alk1+/+ or Alk1+/− mice after the application of a flow of 2 dyne/cm2 for 2 min (n = 3). Results were considered statistically significant if p < 0.05 (ns: non-significant; *: p < 0.05; **: p < 0.01; ***: p < 0.001; ****: p < 0.0001). [file Image_3.JPEG]

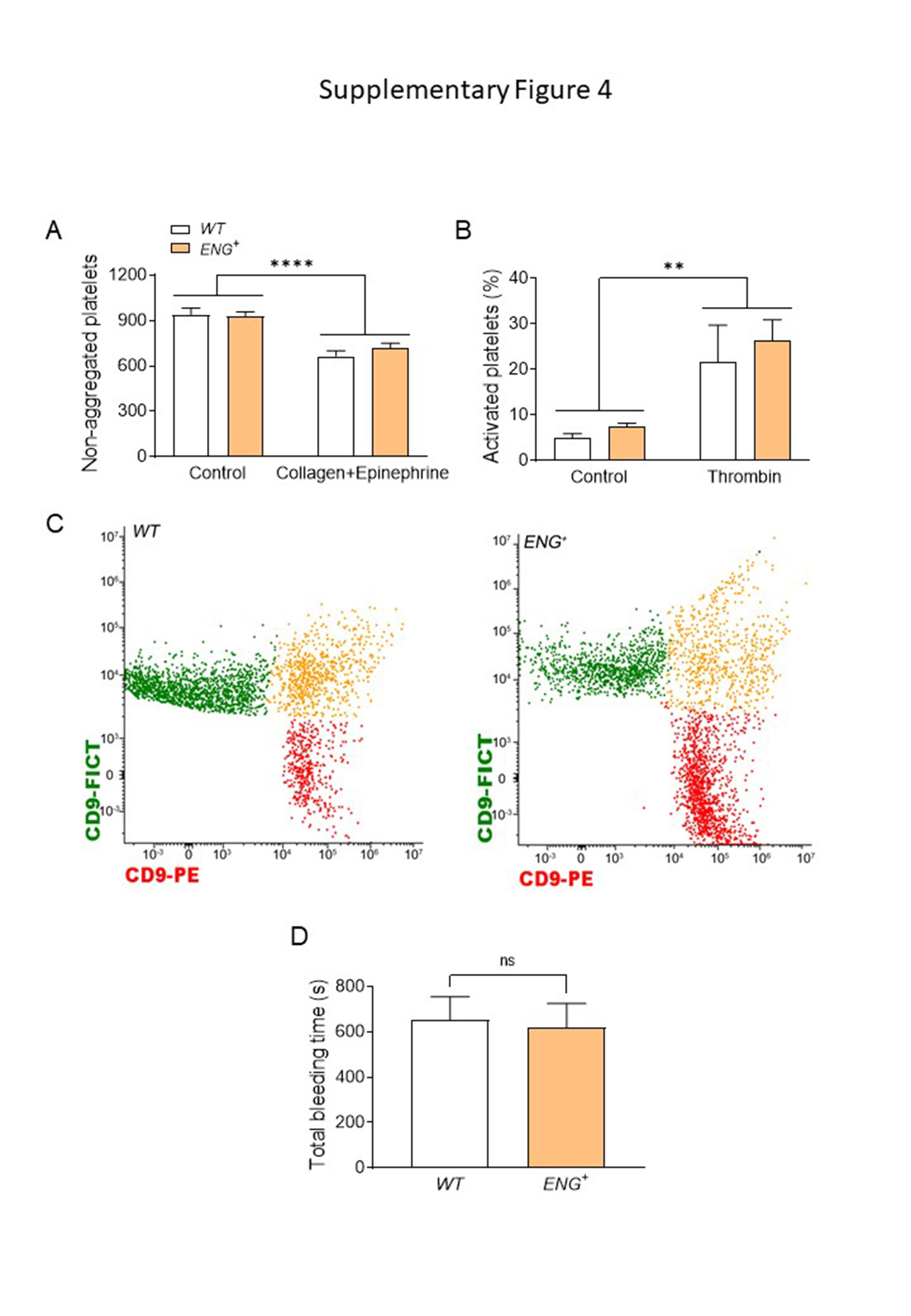

Supplement: Supplementary Figure S4 — (A) Number of non-aggregated platelets per mL before and after the induction of a thrombogenic event with a bolus of collagen and epinephrine in ENG+ mice (n = 17) and its respective WT controls (n = 17). (B) Total bleeding time, calculated as the sum of first bleeding time and rebleeding time, in WT (n = 25) and ENG+ mice (n = 25). (C) Representative images, from 3 independent experiments, of flow cytometry analysis of platelet aggregation after thrombin stimulation of diluted blood from WT and ENG+ mice. Results were considered statistically significant if p < 0.05 (ns: non-significant; *: p < 0.05; **: p < 0.01; ***: p < 0.001; ****: p < 0.0001). [file Image_4.JPEG]
